# Development of Astaxanthin-Loaded Nanosized Liposomal Formulation to Improve Bone Health

**DOI:** 10.3390/ph15040490

**Published:** 2022-04-18

**Authors:** Hsin-I. Chang, Chu-Wen Shao, Evelyn Huang, Kuo-Yuan Huang

**Affiliations:** 1 Department of Biochemical Science and Technology, National Chiayi University, No. 300, Syuefu Rd, Chiayi City 60004, Taiwan; hchang@mail.ncyu.edu.tw (H.-I.C.); wendy44687@yahoo.com.tw (C.-W.S.); 2 Taipei American School, 800 Zhongshan North Road, Section 6, Taipei 11152, Taiwan; eve031017@gmail.com; 3 Department of Orthopedics, National Cheng Kung University Hospital, College of Medicine, National Cheng Kung University, Tainan 70403, Taiwan

**Keywords:** astaxanthin, marine natural product, liposomes, anti-inflammation, osteoblast mineralization

## Abstract

Astaxanthin is a xanthophyll carotenoid commonly found in marine organisms. Due to its super antioxidative ability, astaxanthin has been widely applied as a human nutraceutical supplement for health benefits. In order to enhance the bioavailability of astaxanthin, we used soybean phosphatidylcholine to encapsulate astaxanthin for liposomal formation. The physical properties of astaxanthin (asta)-loaded liposomes were determined by particle size, encapsulation efficiency and polydispersity index. The results revealed that the particle sizes of asta-loaded liposomes with various concentrations exhibited mean diameters in the range of 109 to 134 nm and had a narrow PDI value. As expected, the entrapment efficiency of liposomes loaded with a low concentration of astaxanthin (0.05 μg/mL) was 89%, and that was reduced to 29% for 1.02 μg/mL asta loading. Alizarin red staining and calcium content measurement showed that there was a significant reduction in calcium deposition for 7F2 osteoblasts treated with asta-loaded liposomes (0.25–1.02 μg/mL) in comparison with the cells treated with drug-free liposomes and mineralization medium (MM). Although liposomal formulation can reduce the cytotoxicity of astaxanthin and possess antioxidant, anti-inflammatory and anti-osteoclastogenic activities in RAW264.7 macrophages, asta-loaded liposomes with high concentrations may suppress ALP activity and mineralization level in 7F2 osteoblasts. Therefore, astaxanthin extract may be able to protect bones against oxidative stress and inflammation through liposomal formulation.

## 1. Introduction

**Figure 1:**
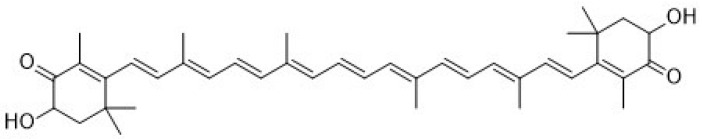
The chemical structure of astaxanthin.

Recently, astaxanthin (3,3′-dihydroxy-β,β-carotene-4,4′dione, Figure 1) has been widely applied as a human nutraceutical supplement for health care or an essential ingredient in the food and cosmetic industry. Astaxanthin is a lipid-soluble reddish pigment belonging to the xanthophyll group of carotenoids, produced by microalgae under pressure such as strong light, high salinity and low nutrient utilization for protection [1]. Astaxanthin could accumulate in the tissues of marine organisms feeding on microalgae. Therefore, astaxanthin is commonly found in green microalgae *Haematococcus pluvialis*; red yeast *Xanthophyllomyces dendrorhous* (*Phaffia rhodozyma*); and marine animals such as shrimp, krill, salmon, crab and lobster. According to numerous studies, astaxanthin showed better biological potential than other antioxidants such as lutein, lycopene, β-carotene and vitamin C [2,3]. Unlike other types of carotenoids, astaxanthin has two keto groups located at the 4,4′ position of the β-ionone ring that activate adjacent hydroxyl groups for capturing per-oxidants and stabilizing the trapped radicals [4]. Through its antioxidant ability, astaxanthin can suppress cancer cell proliferation, migration or invasion; prevent cardiovascular diseases and diabetes; and promote immune system and ocular health [2,5,6,7,8,9]. In addition, astaxanthin has been reported to have several other health benefits, including anti-inflammatory activity, anti-skin-aging ability, protection against early brain injury and neuroprotective property [10,11,12,13,14]. So far, relatively few studies have investigated the association between astaxanthin and bone health [15,16].

Astaxanthin is a partially hydrophobic carotenoid that dissolves in organic solvents such as ethanol, acetone, dimethyl sulfoxide (DMSO) and dimethylformamide (DMF) [17]. As expected, astaxanthin has only slight solubility in water. In addition, astaxanthins in the market (extracted from algae *Haematococcus pluvialis*) are mostly in esterified form, containing various fatty acids [18]. Therefore, these hydrophobic characteristics could limit the clinical application of astaxanthin. In nature, astaxanthin exists mainly in trans-isomeric forms, which could be easily converted into cis-isomeric forms (9-cis-astaxanthin and 13-cis-astaxanthin) due to environmental factors such as heat, light and oxidation [19]. Lin et al., reported that the main astaxanthin components in spear shrimp shells are *trans*-astaxanthin, 9-cis-astaxanthin, 13-cis-astaxanthin and 16 astaxanthin esters [20]. In order to achieve a circular economy, shrimp shell waste is a good source from which to extract astaxanthin. On the other hand, liposomes have been used in the treatment of osteoarthritis, but there is no literature on the evaluation of the effect of astaxanthin-loaded liposomes on bone health. Previous studies have indicated that the biological efficacy of the astaxanthin extracts may change with the manner of storage and extraction [1,21]. In the present study, astaxanthin (asta)-loaded liposomes prepared using soybean phosphatidylcholine (SPC) were expected to maintain the bioactivity of the astaxanthin extract and increase the absorption rate in the bone cells. Therefore, the aim of this study is to provide a new approach to bone health supplementation and increase the economic value of shrimp shell waste through the formulation of asta-loaded liposomes.

## 2. Results

### 2.1. Antioxidant Capacity of Astaxanthin Extract

**Figure 2:**
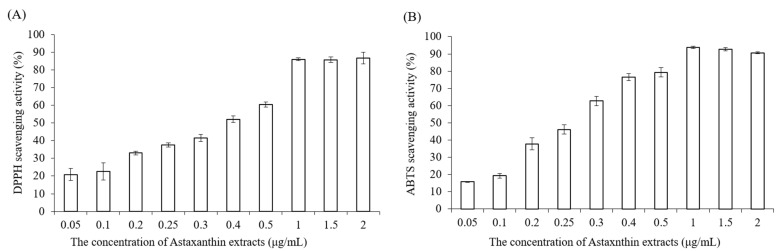
The antioxidant ability of astaxanthin extract measured by DPPH (**A**) and (**B**) ABTS assays.

DPPH and ABTS assays are analytical techniques extensively used for determining the antioxidant activity of natural products. Here, the antioxidant capacities of astaxanthin extract at various tested concentrations (0.05, 0.1, 0.2, 0.25, 0.3, 0.4, 0.5, 1, 1.5, 2 μg/mL) were detected by DPPH and ABTS methods. According to both DPPH (Figure 2A) and ABTS assays (Figure 2B), the scavenging antioxidant capacity of astaxanthin was increased in a dose-dependent manner. In particular, the radical scavenging capacity reached around 80% when the concentration of the astaxanthin extract was 1 μg/mL. Therefore, we confirmed that the astaxanthin extract exhibited a powerful antioxidant ability. Although astaxanthin extract has strong antioxidant properties, cytotoxic effects may be induced by overdose, and hence it is important to find suitable doses and determine the toxicity limit of the astaxanthin extract. Thus, our results (Figure 2A) showed that low doses of astaxanthin extract (0.05~0.25 μg/mL) still have 20~37% of DPPH antioxidant activity, and these doses were selected for use in further experiments. The concentrations of 1 and 2 μg/mL were chosen for presentation as high doses of the astaxanthin extract.

### 2.2. Characterization of Liposomal Formulations

The drug delivery capability was closely related to the physical properties of liposomes. Therefore, evaluating the physicochemical characterization of liposomal formulations, including particle size, polydispersity index (PDI), entrapment efficiency (EE) and liposomal stability, was a crucial point once the astaxanthin extract (asta)-loaded liposomes were produced. Table 1 demonstrates that the particle size of empty liposomes was around 134.35 nm and asta-loaded liposomes with various loading concentrations exhibited mean diameters in the range of 109 to 128 nm. Therefore, the liposomal encapsulation of astaxanthin extract could slightly reduce particle size. PDI plays an important role in managing the physical stability of liposomes [22]. As expected, empty and asta-loaded liposomes had a narrow PDI value, and the value increased in a dose-dependent manner for asta-loaded liposomes. Moreover, EE is an expression of the amount of drug incorporated into the liposome and is considered a major factor in obtaining desired therapeutic results [23]. However, EE showed a decline from 89% to 29% when liposomes were loaded with asta at concentrations from 0.05 to 1.02. Based on the above results, asta-loaded liposomes could increase the PDI value and reduce EE with an increase in asta loading concentration.

### 2.3. Physical Stability of Asta-Loaded Liposomes in Storage

**Figure 3:**
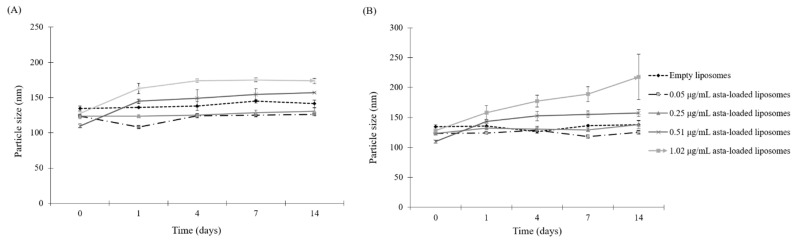
Physical stabilities of liposomal formulations. (**A**) Asta-loaded and empty liposomes were stored at 4 °C in DMEM containing 10% FBS over 1, 4, 7 and 14 days of incubation. (**B**) Asta-loaded and empty liposomes were stored at 37 °C in DMEM containing 10% FBS and incubated for 1, 4, 7 and 14 days. The particle sizes are presented as the mean ± standard deviation.

Good storage is critical in maintaining the shelf-life stability of the asta-loaded liposomes, including the uniformity of size distribution and encapsulation efficiency and minimal degradation of all compounds [24]. In order to know whether serum will cause aggregation with the liposomes, storage stability studies were carried out by measuring the mean vesicle particle size during 14 days of incubation at 4 °C and 37 °C. As shown in Figure 3A,B, the mean diameters of empty and asta-loaded liposomes with low doses (0.05 and 0.25 μg/mL) were not significantly different during 14 days of incubation at 4 °C. On the other hand, the average diameter of asta-loaded liposomes with high doses (0.51 and 1.02 μg/mL) presented an increasing tendency in particle size. As shown in Figure 3B, the mean diameter of 1.02 μg/mL asta-loaded liposomes was increased up to 217 nm after 14 days of incubation at 37 °C. These results revealed that the high doses of asta loading and high storage temperature could induce the liposomal aggregations with serum. Therefore, asta loading concentration and storage temperature could play important roles in the physical stability of asta-loaded liposomes.

### 2.4. The Cell Uptake of DiI-Labeled Liposomes in 7F2 Osteoblasts

**Figure 4:**
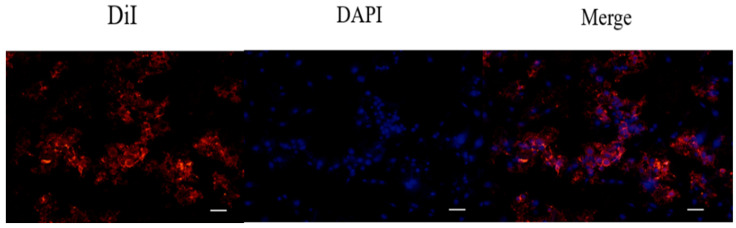
In vitro uptake of DiI-labeled liposomes in 7F2 osteoblasts measured by fluorescent microscopy. The cells were cultured with DiI-labeled liposomes for 4 h. Thereafter, red fluorescence (DiI-labeled liposomes) and blue fluorescence (DAPI for nuclei staining) were evaluated by fluorescent microscopy equipped with a CCD system (×100 magnification, scale bar = 200 nm).

To investigate the cell uptake of the liposomes, 7F2 osteoblasts were treated with DiI-labeled liposomes for 4 h and subsequently analyzed by fluorescent microscopy (Figure 4). The aim of this procedure was to evaluate whether liposomes could act as effective vehicles for the target cells. According to the red fluorescent liposomes surrounding cells in blue fluorescence (nuclei staining), we could confirm that DiI-labeled liposomes were successfully delivered into cells. 

### 2.5. The Effect of Astaxanthin and Asta-Loaded Liposomes on Cell Viability of Raw264.7 Mouse Macrophages

**Figure 5:**
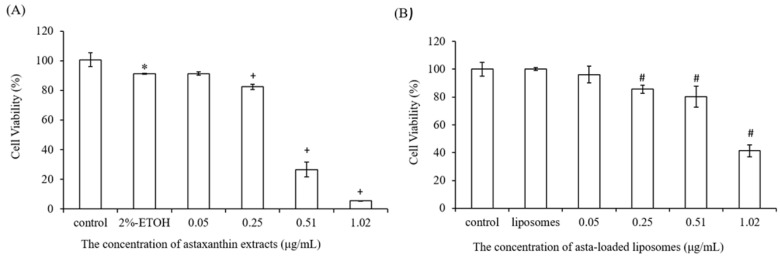
The effect of astaxanthin extract and asta-loaded liposomes on cell viability of Raw264.7 mouse macrophages. Macrophages were treated with (**A**) astaxanthin extract and (**B**) empty and asta-loaded liposomes for 24 h. Viabilities of Raw264.7 macrophages were measured by MTT assay. The data are shown as the means ± standard deviation. (* *p* < 0.05 related to control; ^+^ *p* < 0.05 related to 2% ETOH; ^#^ *p* < 0.05 related to liposomes).

As shown in Figure 5A, the viability of Raw264.7 mouse macrophages was monitored after 24 h incubation with different concentrations of astaxanthin extract. Because astaxanthin extract can be dissolved in ethanol, ethanol was used as a control group. The result presented that cell viability was reduced in a dose-dependent manner for the cells treated with astaxanthin extract, especially at high doses (0.51 and 1.02 μg/mL). When the cells were exposed to 0.51 and 1.02 μg/mL concentrations of astaxanthin extract, there were about 74% and 95% reductions in cell viability. Next, we measured cell viability for Raw264.7 mouse macrophages treated with asta-loaded liposomes. As shown in Figure 5B, high doses of asta-loaded liposomes (0.51 and 1.02 μg/mL) can improve the viability of Raw264.7 macrophages from 26% to 80% and from 5% to 41% in comparison with cells treated with astaxanthin extract. As expected, asta-loaded liposomes also dose-dependently reduced the cell viability of Raw264.7 macrophages. Based on these experimental results, the cytotoxicity in Raw264.7 macrophages was related to the dose of astaxanthin extract, but liposomal formulation could reduce the cytotoxicity of astaxanthin extract. The doses of asta-loaded liposomes which can maintain 80% of cell viability in Raw264.7 macrophages were further studied for anti-inflammatory activities.

### 2.6. The Effect of Astaxanthin and Asta-Loaded Liposomes on Cell Viability of 7F2 Osteoblast Cells

**Figure 6:**
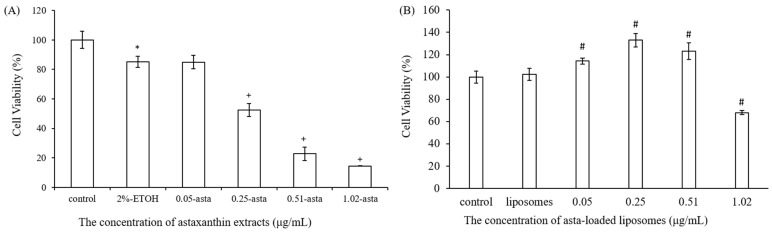
The effect of astaxanthin extract and asta-loaded liposomes on cell viability of 7F2 osteoblasts. 7F2 osteoblasts were treated with (**A**) astaxanthin extract and (**B**) empty and asta-loaded liposomes for 24 h. Cell viabilities of 7F2 osteoblasts were measured by MTT assay. The data are shown as the means ± standard deviation. (* *p* < 0.05 related to control; ^+^ *p* < 0.05 related to 2% ETOH; ^#^ *p* < 0.05 related to liposomes).

We also evaluated the cytotoxicity of astaxanthin extract and asta-loaded liposomes on 7F2 osteoblasts. As shown in Figure 6A, the inhibitory effect of astaxanthin extract on the cell viability of 7F2 osteoblasts was dose-dependent. Furthermore, Figure 6B shows that cells treated with 0.05 to 0.51 μg/mL of asta-loaded liposomes had over 100% cell viability after 24 h, and hence asta-loaded liposomes may slightly induce cell proliferation of 7F2 osteoblasts. Although cells treated with 1.02 μg/mL of asta-loaded liposomes only showed about 40% cell viability, they still presented higher cell viability than the cells treated with astaxanthin extract at the same concentration. Interestingly, 7F2 osteoblasts treated with 0.25 and 0.51 μg/mL asta-loaded liposomes can exhibit increases in cell number by up to 132% and 123%, respectively. Therefore, the liposomal formulation of astaxanthin extract has a major influence on reducing the cytotoxicity of 7F2 osteoblasts and may have the potential to stimulate cell proliferation. The doses of asta-loaded liposomes which can maintain 80% of cell viability in 7F2 osteoblasts were further studied for osteoblast differentiation and mineralization.

### 2.7. The Effect of Asta-Loaded Liposomes on Nitrite Production, COX-2 Expression and TRAP Activity in LPS-Induced Raw264.7 Mouse Macrophage Cells

**Figure 7:**
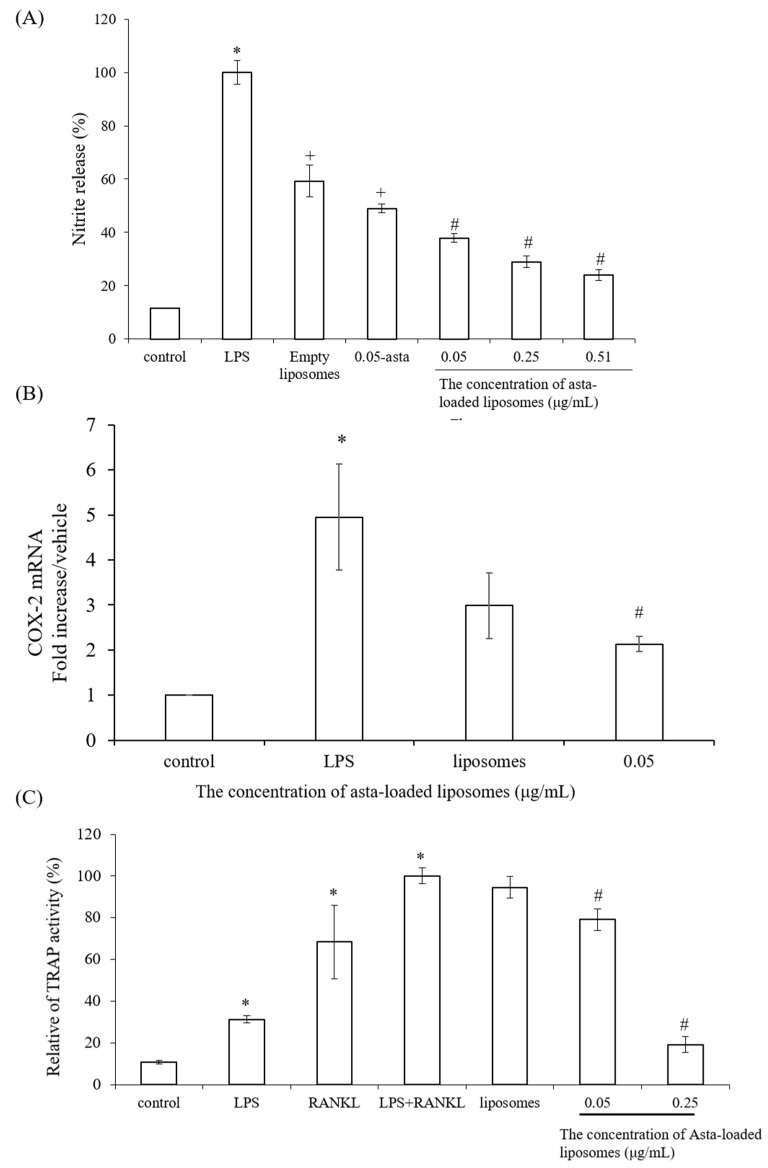
The effects of asta-loaded liposomes on nitrite production, Cox-2 expression and TRAP activity of LPS-induced Raw264.7 mouse macrophages. Raw264.7 macrophages were co-treated with LPS and asta-loaded liposomes overnight. (**A**) NO production in no-phenol red medium was evaluated by chemical Griess reagent. Nitric oxide assay is a colorimetric method, once the Griess reagents contact the solution containing nitrite ions, the reaction mixture will change color to pink; the absorbance was measured at OD 550 nm. The nitrite production in LPS-induced cells is presented as 100% (* *p* < 0.05 related to control, ^+^ *p* < 0.05 related to LPS and ^#^ *p* < 0.05 related to cells treated with empty liposomes). (**B**) COX-2 mRNA expression of Raw264.7 macrophages. Real-time PCR analysis was performed in Raw264.7 macrophages after 24 h treatment with LPS (* *p* < 0.05 related to control and ^#^ *p* < 0.05 related to LPS). (**C**) Asta-loaded liposomes inhibit TRAP activity in LPS- and RANKL-induced RAW264.7 macrophages. Data are expressed as the percentage of TRAP activity measured in cells derived from each model in comparison with control (* *p* < 0.05 related to control and ^#^ *p* < 0.05 related to cells treated with LPS and RANKL).

In order to evaluate the anti-inflammatory effects of asta-loaded liposomes, NO production and COX-2 expression were determined in LPS-stimulated Raw264.7 macrophages. Figure 7A indicated that NO production in LPS-stimulated macrophages was about 9-fold higher than that in the control. When LPS-induced macrophages were co-treated with asta-loaded liposomes, NO production was significantly inhibited in a dose-dependent manner. In addition, the reduction in NO production for the cells treated with 0.05 μg/mL asta-loaded liposomes (63%) was higher than that observed in cells treated with astaxanthin extract (52%) at the same concentration. The suppressive effect of asta-loaded liposomes on the COX-2 expression in LPS-activated macrophages was further analyzed. As shown in Figure 7B, empty and asta-loaded liposomes caused a decrease in the LPS-mediated induction of COX-2. Asta-loaded liposomes are more effective at suppressing COX-2 expression than empty liposomes. TRAP activation is essential for the osteoclast formation in macrophages by LPS and NANKL, and asta-loaded liposomes are effective at inhibiting NO production and COX-2 expression by LPS. Next, we investigated whether or not asta-loaded liposomes could suppress TRAP activities in LPS- and RANKL-induced RAW264.7 macrophages. The induction of TRAP activity by LPS and RANKL was markedly inhibited by asta-loaded liposomes in a dose-dependent manner (Figure 7C). Therefore, our results suggest that the anti-osteoclastogenic effect of asta-loaded liposomes was mainly exerted by the inhibition of LPS-induced COX-2 expression and NO production. 

### 2.8. The Effect of Asta-Loaded Liposomes on Intracellular ROS Production in LPS-Induced Raw264.7 Mouse Macrophage Cells

**Figure 8:**
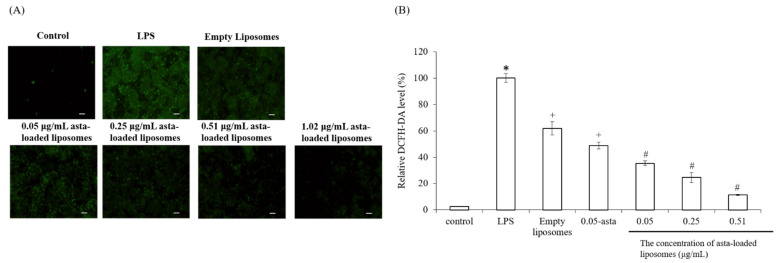
The effect of asta-loaded liposomes on intracellular ROS production of LPS-induced Raw264.7 mouse macrophages. Raw264.7 macrophages were co-treated with LPS and asta-loaded liposomes overnight. (**A**) Production of reactive oxygen species (ROS) (×100 magnification, scale bar = 200 nm). (**B**) Quantification of relative fluorescence intensity. Results are revealed as percentages with mean ± standard deviation (*n* = 4–6). Fluorescence images and quantitation of relative fluorescence intensity representing cytosolic ROS detection by DCF-DA staining (* *p* < 0.05 related to control, ^+^ *p* < 0.05 related to LPS and ^#^ *p* < 0.05 related to cells treated with empty liposomes).

It is reported that LPS-induced inflammation is related to the reactive oxygen species (ROS)-induced oxidative stress. In order to determine whether asta-loaded liposomes can inhibit inflammatory response by reducing ROS levels in LPS-induced Raw264.7 mouse macrophages, we used DCF-DA fluorescent staining to measure intracellular ROS production. As shown in Figure 8, intracellular ROS production in Raw264.7 macrophages was increased about 50 times higher than that in the control through LPS stimulation. Due to the diminishing of DCF-DA fluorescent expressing cells, we confirmed that asta-loaded liposomes can dose-dependently reduce the cellular oxidative stress. At the same concentration, the ROS level in the cells treated with 0.05 μg/mL asta-loaded liposomes (35%) was lower than that in cells treated with astaxanthin extract (48%). Thus, asta-loaded liposomes could protect Raw264.7 cells from LPS-induced oxidative stress by scavenging intracellular ROS.

### 2.9. The Influence of Asta-Loaded Liposomes on 7F2 Osteoblast Differentiation and Mineralization

**Figure 9:**
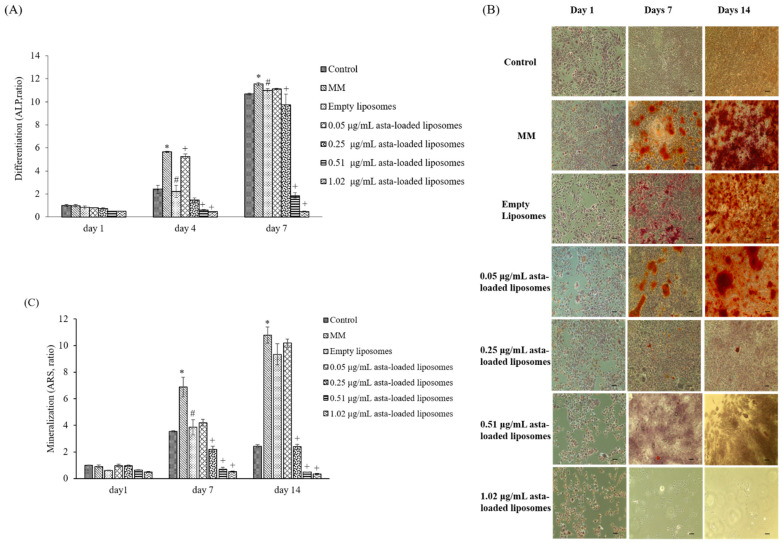
The effect of asta-loaded liposomes on 7F2 osteoblast differentiation and mineralization. 7F2 osteoblasts were treated with 50 μg/mL of ascorbic and 10 mM β-glycerophosphate to induce osteoblast differentiation and mineralization. (**A**) Cells were then incubated in the presence or absence of asta-loaded liposomes for 1, 4 and 7 days. Results are revealed as a ratio with mean ± standard deviation (*n* = 3). (**B**) ARS staining of calcium deposits on days 1, 7 and 14 (×100 magnification, scale bar = 200 nm). (**C**) Quantification of calcium deposits on days 1, 7 and 14. Calcium deposition was quantified by dissolving ARS aggregates into 10% cetylpyridinium chloride and evaluating the absorbance at 560 nm. Results are revealed as a ratio with mean ± standard deviation (*n* = 3) (* *p* < 0.05 related to control group of the same day, ^#^ *p* < 0.05 related to MM of the same day, ^+^ *p* < 0.05 related to empty liposome treatment of the same day).

Alkaline phosphatase (ALP) is a specific key component in osteoblast differentiation and mineralization, and hence we investigated the effect of asta-loaded liposomes on 7F2 osteoblasts by measuring ALP activity. Osteoblasts were incubated with mineralization medium (MM) and co-treated with asta-loaded liposomes for 1 to 7 days. Figure 9A reveals a time-dependent increase in ALP activities in 7F2 osteoblasts, except for the cells treated with high-dose asta-loaded liposomes (0.51 and 1.02 μg/mL). It is worth noting that cells treated with 0.05 μg/mL asta-loaded liposomes showed better ALP activity than ones treated with empty liposomes, but there was no significant difference on day 7. 

The influence of asta-loaded liposomes on osteoblast mineralization was analyzed by ARS staining assay after 1, 7 and 14 days of incubation. ARS staining (Figure 9B) showed mineralization deposits (red color) in cells treated with MM, empty or 0.05 μg/mL of asta-loaded liposomes after 7 and 14 days of incubation. In comparison with the control, there was a 4-fold increase in mineralization level for the cells treated with empty or 0.05 μg/mL of asta-loaded liposomes on day 14 (Figure 9C). However, there was no significant difference in the mineralization level for the cells treated with empty or 0.05 μg/mL of asta-loaded liposomes. Cells treated with 0.25, 0.51 and 1.02 μg/mL of asta-loaded liposomes revealed a dramatic reduction in osteoblast mineralization after 7 and 14 days of incubation. Taking all the results together, 7F2 osteoblasts treated with 0.05 μg/mL of asta-loaded liposomes showed similar ALP activity and mineralization level to the cells treated with MM only, and hence 0.05 μg/mL could be the best concentration of asta-loaded liposomes for osteoblast differentiation and mineralization.

## 3. Discussion

Since astaxanthin has low aqueous solubility, this limitation could be a significant barrier in the application of pharmaceutical or health products, and that can be overcome by the microencapsulation of the liposome. The therapeutic efficiency of the natural extracts such as Fraxinus angustifolia leaf and bark extracts can be improved by liposomal encapsulation techniques [24]. In this study, SPC liposomes were formulated in order to enhance the biological activities of astaxanthin extracts. In the physical properties of asta-loaded liposomes, there were no significant differences in particle sizes, but the PDI value was raised by increasing the concentration of the astaxanthin extract. Similar to the research of David R. Khan et al., storage stability experiments demonstrated that liposomal formulations of astaxanthin extracts were more stable in the storage under refrigeration temperature, and asta-loaded liposomes with high concentrations will be less stable [25]. We also found that liposomes reduced the entrapment efficiency of astaxanthin from 89% to 29% when the concentration of astaxanthin extract was increased from 0.05 to 1.02 μg/mL. The asta-loaded liposomes were less toxic in vitro than the free drug, and that may promote osteoblast proliferation.

To confirm the powerful antioxidant, anti-inflammatory and anti-osteoclastogenic potential of astaxanthin, we performed a scavenging intracellular ROS assay, nitrite production assay, TRAP activity assay and RT-PCR analysis. Lee et al., have mentioned that astaxanthin can inhibit the expression of iNOS, COX-2, TNF-α and IL-1β as well as the production of NO and PGE2 in LPS-induced macrophages [26]. Our results also demonstrated that asta-loaded liposomes can exert anti-inflammatory effects via the suppression of COX-2 and NO. Oxidative stress and inflammation are closely related pathological processes, and hence inflammatory mediators and cytokines can accelerate intracellular ROS accumulation. In our study, asta-loaded liposomes were found to protect RAW264.7 macrophages against LPS-stimulated oxidative stress by reducing intracellular ROS accumulation. Similar to our results, Liu et al., indicated that astaxanthin could significantly suppress ROS production induced by 6-hydroxydopamine (6-OHDA), which may cause Parkinson’s disease [27]. Therefore, we might confirm that asta-loaded liposomes can reduce oxidative damage caused by excessive ROS or inflammation.

In previous studies reported by our lab, mineralization medium (MM, 5 mM β-glycerophosphate and 50 μg/mL ascorbic acid) was able to induce osteoblast differentiation and mineralization in 7F2 osteoblast-like cells [28]. In comparison with cells treated with MM only, 0.05 μg/mL asta-loaded liposomes showed a similar pattern in osteoblast differentiation and mineralization. Similar to our finding, Zhang and Peng (2019) demonstrated that 20 ng/mL astaxanthin-encapsulated polymeric micelles could enhance osteogenic differentiation of human mesenchymal stem cells [29]. Moreover, Hwang et al., indicated that astaxanthin may inhibit osteoclast formation through the expression of the nuclear factor of activated T cells (NFAT-c1), dendritic cell-specific transmembrane protein (DC-STAMP), TRAP and cathepsin K [16]. Consistent with previous results, asta-loaded liposomes could suppress TRAP activities in LPS- and RANKL-induced RAW264.7 macrophages through anti-inflammatory and antioxidant pathways. In addition, El-Baz et al., demonstrated that *Heamatococcus pluvialis* microalgae, containing astaxanthin, could ameliorate bone loss through the downregulation of serum OPG and upregulation of serum RANKL [15]. Therefore, asta-loaded liposomes with low doses could inhibit osteoclastogenesis through the inhibition of TRAP activities in concurrence with no inhibitory effect on osteoblast proliferation and differentiation.

Astaxanthin is a valuable functional ingredient that has the ability to resist ROS accumulation and suppress oxidative stress associated with inflammation. Liposomal formulation of astaxanthin extract can reduce the cytotoxicity of free drugs, and asta-loaded liposomes with a low dose (0.05 μg/mL) can retain osteoblastic activity. Thus, we suggested that asta-loaded liposomes could be applied as an antioxidant and anti-inflammatory ingredient as well as a keeper of osteoblastic mineralization in nutritional health products. 

## 4. Materials and Methods

### 4.1. Materials

Astaxanthin extract from dried shrimp heads was purchased from Acorty Biotechnology Co., Ltd., Chia Yi City, Taiwan. 2,2-Diphenyl-1-picrylhydrazyl (DPPH), alizarin red S (ARS) and thiazolyl blue tetrazolium bromide (MTT) were purchased from Sigma-Aldrich, Saint Louis, MO, USA. Alkaline Phosphatase Assay kit (colorimetric) was acquired from Abcam (Cambridge, UK). ABTS was purchased from Calbiochem (San Diego, CA, USA). All of the cell culture materials were acquired from Gibco (Grand Island, NT, USA), and all solvents used were of analytical grade (J.T. Baker, Phillipsburg, NJ, USA).

### 4.2. Cell Culture

Mouse osteoblast-like cells (7F2) and mouse macrophage cells (Raw264.7) were obtained from Bioresource Collection and Research Center (BCRC), Taiwan. 7F2 and Raw264.7 cells were cultured in Dulbecco’s modified Eagle’s medium (DMEM, Gibco Thermo Fisher Scientific, Inc., Waltham, MA, USA) containing 10% fetal bovine serum (FBS), 1% 100 units/mL penicillin and 100 μg/mL of streptomycin and 1% 200 mM L-glutamine. Cells were maintained at 37 °C with 5% CO_2_ in a humidified incubator and subcultured at an initial density of 5 × 10^5^/mL every 2–3 days.

### 4.3. DPPH Scavenging Antioxidant Activity

Astaxanthin extract was diluted with ethanol to make various concentrations (0.05, 0.1, 0.2, 0.25, 0.3, 0.4, 0.5, 1, 1.5, 2 μg/mL). Briefly, 0.25 mM 2,2-diphenyl-1-picrylhydrazyl (DPPH) was prepared with methanol before the measurement, the blank solution was sample solvent (ethanol, 100 μL), and 100 μL DPPH reagent was mixed with 100 μL ethanol to serve as a control. Next, 100 μL astaxanthin samples and 100 μL DPPH reagent were added to 96-well plates, shaken gently and incubated for 20 min in the dark at room temperature. Finally, the absorbance of mixture solutions was determined at 517 nm by using an ELISA reader (Tecan, Infinite M200). The measurements were performed in triplicate. The radical-scavenging activity was expressed as *percentage* of inhibition (AS %) and calculated by the following equation:

$$\mathrm{AS}\;\%=\left[1-\frac{ABS\;sample-ABS\;blank}{ABS\;control}\;\right]\times 100\%$$

### 4.4. ABTS Radical Scavenging Activity

The 2,2-azinobis (3-ethylbenzothiazoline-6-sulfonic acid) (ABTS) assay determines the scavenging ability of antioxidant activity by reaction with a strong antioxidant agent (potassium permanganate or potassium persulfate) in the presence of ABTS salt [30]. The ABTS stock solution was prepared by mixing 7 mM ABTS aqueous solution with 2.45 mM aqueous solution of potassium peroxodisulfate in equal quantities and allowed to react at room temperature in the dark for 12–16 h. To make sure the working solution was able to be used in the further experiment, the stock solution was diluted with distilled water, and the absorbance was measured at 734 nm by using an ELISA reader (Tecan, Infinite M200). The absorbance value must be in the range of 0.7 ± 0.02. Astaxanthin extract was diluted with ethanol to prepare various concentrations (0.05, 0.1, 0.2, 0.25, 0.3, 0.4, 0.5, 1, 1.5, 2 μg/mL). Then, 40 μL astaxanthin samples and 160 μL ABTS working solution were added to 96-well plates carefully, and the mixtures were shaken tenderly and incubated in the dark at 37 °C for 10 min. Finally, the absorbance of the mixture solutions was determined at 734 nm by using an ELISA reader (Tecan, Infinite M200).

### 4.5. Liposomal Formulation

The preparation of astaxanthin-loaded liposomes was performed based on the modified thin-film hydration method [31]. Ong et al., demonstrated that liposomes prepared using chloroform possessed better encapsulation efficiency [23]. Therefore, chloroform was chosen as the solvent for the preparation of astaxanthin-loaded liposomes. First, 100 mg of phospholipids was dissolved in 8 mL of chloroform. Next, different amounts of astaxanthin were dissolved in 2 mL of ethanol. Later, both phospholipid and astaxanthin solutions were mixed together in a round-bottom flask. The organic solvent of the astaxanthin/phospholipid mixtures was evaporated by a rotary evaporator (Eyela, N-1000, Tokyo, Japan) at 45 °C and then vacuum-dried to form a lipid film. Next, 2 mL phosphate-buffered saline was used to rehydrate the dry lipid film to form liposomes. Finally, the liposomes were downsized by sequence passing through polycarbonate membranes with pore sizes of 400 nm and 200 nm using an extruder (Avanti Mini-Extruder, Alabaster, AL, USA) for uniform and reduced particle size. Empty liposomes were prepared by the same procedure but only with drug-free ethanol.

### 4.6. Particle Characterization

The particle size of liposomes was measured using a dynamic light scattering instrument (LB-550, Horiba Ltd., Kyoto, Japan). Liposomal dispersions were diluted with double-distilled water at the ratio of 5:1 in cuvettes to guarantee the light scattering intensity in the instrument’s sensitivity range. The particle size and polydispersity index (PDI) of liposomes were measured immediately after liposomal extrusion. The liposomes were incubated with culture medium at the ratio of 1:10 at different temperatures (4 °C and 37 °C) for 1, 4, 7 and 14 days. All measurements were taken in triplicate. The polydispersity index (PDI) was calculated by the following equation according to the average value of the particle size:

$$\mathrm{PDI}={\left(S.D÷mean\;size\right)}^{2}$$

### 4.7. Entrapment Efficiency

Once the liposomes were made, high-speed centrifugation was used to analyze the amount of astaxanthin loaded in liposomes. Astaxanthin-loaded liposomes were spun at 80,000 rpm for about 30 min using a Beckman ultra-high centrifuge. Then, the supernatants which contained unentrapped astaxanthin were carefully withdrawn. Next, the pellets were dissolved with the same volume of ethanol, and absorbance was measured at 480 nm, which is one of the major absorbance peaks of astaxanthin, by an ELISA reader (Tecan, Infinite M200). The entrapment efficiency of astaxanthin in liposomes was calculated by the standard curve. The entrapment efficiency (EE) was estimated using the following equation:

$$\mathrm{EE}\%\;=\;\frac{The\;amount\;of\;astaxanthin\;in\;liposomes}{Initial\;amount\;of\;asatxanthin\;for\;drug\;loading}\times 100\%$$

### 4.8. Determination of Cell Uptake of DiI-Labeled Liposomes in 7F2 Osteoblasts by Fluorescence Staining

After liposomal formulation, a lipophilic solution of DiI (1,1′-dioctadecyl-3,3,3′3′-tetramethylindocarbocyanine perchlorate, St Louis, MO, USA) was used for the fluorescent staining in order to investigate cell uptake of astaxanthin-loaded nanoparticle liposomes. First, 1 μL of DiI stock solution (10 mg of DiI powder dissolved in 1 mL of ethanol) was added to the phospholipid solution to form DiI-loaded liposomes. Briefly, 7F2 osteoblast-like cells were seeded in a 3.5 cm dish at a density of 5 × 10^4^ cells/dish. After 24 h, the cell culture medium was replaced with a basal medium containing DiI-loaded liposomes, and cells were incubated for 4 h. Later, 4% formaldehyde was used to fix the cells for 30 min, and the cells were then rinsed with PBS and stained with DAPI dye (2-(4-amidinophenyl)-1H-indole-6-carboxamidine, 10 μg/mL) for 10 min. After washing with PBS twice, the cells were soaked with 1 mL PBS and photographed using a microscope (Nikon TI-E) and a CCD camera system (SPOT RT3). The photographs were quantified by ImageJ software.

### 4.9. Cell Viability and Proliferation Assay

A stock thiazolyl blue tetrazolium bromide (MTT, Sigma-Aldrich, USA) solution (5.0 mg/mL in phosphate-buffered saline (PBS) was prepared immediately prior to use and filtered through a 0.22 m Millipore filter (Burlington, MA, USA). The MTT solution was stored in 1.5 mL centrifuge tubes in the dark at −20 °C. Briefly, 7F2 osteoblast-like cells and Raw264.7 mouse macrophage cells were seeded in 96-well plates at a density of 1 × 10^4^ cells/well and 5 × 10^4^ cells/well, respectively. After seeding, cells were treated with DMEM medium containing astaxanthin and asta-loaded liposomes at various concentrations for 24 h at 37 °C with 5% (*v*/*v*) CO_2_. Next, the cell supernatants were withdrawn and 100 μL of MTT (3-(4,5-dimethylthiazol-2-yl)-2,5-diphenyltetrazolium bromide) solution was added to each well for 4 h of incubation. Once the MTT reagent was removed, the formazan product was dissolved in 200 μL dimethyl sulfoxide (DMSO), and the absorbance was measured at 570 nm with an ELISA reader (Tecan, Infinite M200). The measurements were performed in quadruplicate, and cell viability was expressed as a percentage of formazan absorbance compared to the control.

### 4.10. Determination of Anti-Inflammatory Activity by Nitrite Assay

The nitrite production in Raw264.7 macrophage cells was measured by Griess reagent. Briefly, mouse macrophage Raw264.7 cells were seeded in 24-well plates at a density of 4 × 10^5^ cells/well and cultured at 37 °C with 5% CO_2_ (*v*/*v*) overnight. Next, media were withdrawn and cells were treated with 1 mL of culture medium containing various concentrations of astaxanthin extract in the presence of lipopolysaccharide (LPS, 0.5 μg/mL) for 24 h of incubation. Cells treated with culture medium and 0.5 μg/mL of LPS were indicated as positive control. After removing supernatants, 400 μL of no-phenol red medium was added for 6 h of incubation. NO release from LPS-induced macrophages was analyzed by determining nitrite concentration. Later, 100 μL aliquots of nitrite-containing supernatants were mixed with the same volume of Griess reagent in 96-well plates and gently shaken in the dark at room temperature for 15 min. To quantify the nitrite concentration, the absorbance of the mixture solutions was evaluated using an ELISA reader (Tecan, Infinite M200) at a wavelength of 550 nm. The data were shown as the mean percentage of absorbance in comparison with the LPS-treated group.

### 4.11. Osteoclast Differentiation Assay

TRAP activity was determined by para-nitrophenylphosphate (pNPP) according to the microplate assay method of Park et al., with the following modifications [28]: Cells were fixed with 10% *w*/*v* formaldehyde for 1 min and then treated with an equal mixture of acetone and formalin for another minute. After desiccation, cells were incubated with 100 μL phosphate substrate solution (3.7 mM pNPP and 10 mM sodium tartrate in 50 mM citrate butter, pH 4.6) at 37 °C for 10 min. Then, the enzyme reaction was stopped by the addition of 100 μL 0.1 N sodium hydroxide solution, and the absorbance of the resulting yellow color product was measured by an ELISA reader at a wavelength of 405 nm.

### 4.12. Detection of Intracellular Reactive Oxygen Species (ROS) by DCF-DA Staining

Cellular ROS levels can be evaluated in live cells by a technique that converts 2′,7′-dichlorofluorescin diacetate (DCF-DA) to a high fluorescence dye (green), 2′,7′-dichlorofluorescein (DCF), upon oxidation. 2′,7′-Dichlorofluorescin diacetate (DCF-DA, Cayman Chemical, Ann Arbor, MI, USA) stock solution was dissolved in ethanol at a concentration of 5 mg/mL and further diluted with PBS saline to 25 μM prior to use. Briefly, Raw264.7 mouse macrophages were seeded in 24-well plates at a density of 1.5 × 10^5^ cells/well. After 24 h, cells were treated with LPS and various concentrations of asta-loaded liposomes (0.05, 0.25, 0.51 μg/mL). Cells were then washed twice with PBS, and fixed with 4% paraformaldehyde for 30 min in dark. Thereafter, cells were rinsed again with PBS, and 25 μM of DCF-DA solution was added to each well for 1 h of staining. Finally, the cells were soaked with PBS and photographed using a microscope (Nikon TI-E) and a CCD camera system (SPOT RT3). The related fluorescence DCF-DA intensity was quantified by ImageJ. Data were shown as the mean percentage in comparison to LPS-induced cells.

### 4.13. Cellular Alkaline Phosphatase (ALP) Assay

The ALP activity was assessed using a colorimetric alkaline phosphatase assay kit. Firstly, the ALP detection reagent was prepared by mixing Alkaline Phosphatase Blue Microwell Substrate Component A&B (SIGMA-ALDRICH) at the ratio of 1:1 at room temperature. Briefly, 7F2 mouse osteoblast-like cells were seeded in 24-well plates at a density of 1 × 10^4^ cells/well. In this experiment, 7F2 osteoblasts were cultivated with mineralization medium (DMEM medium containing 10% FBS, 1% penicillin–streptomycin, 1% L-glutamine, 5 mM β-glycerophosphate and 50 μg/mL ascorbic acid). Then, the cells were treated with the mineralization medium containing various concentrations of asta-loaded liposomes and incubated for 1, 4 and 7 days at 37 °C with 5% CO_2_ (*v*/*v*) atmosphere. Subsequently, the media were withdrawn and cells were rinsed with PBS carefully. Next, 300 μL of 1% Triton-X100 lysis buffer was added and incubated at 37 °C with 5% CO_2_ (*v*/*v*) atmosphere for 10 min to lyse the cells. The cell culture supernatants were moved into the 1.5 mL microtubes and centrifuged at 10,000 rpm for 5 min. Later, 100 μL aliquots of the supernatants were transferred to the 96-well plates, and the same volume of the ALP detection reagent was added to each well to react for 15 min in the dark. The absorbance was measured by an ELISA reader (Tecan, Infinite M200) at a wavelength of 560 nm. The measurements were taken in triplicate.

### 4.14. Mineralization of the Extracellular Matrix

Calcium content measurement and alizarin red S (ARS) staining were performed to assess the mineralization process of 7F2 osteoblasts on days 1, 7 and 14 after cells were treated with asta-loaded liposomes. Briefly, 7F2 osteoblast cells were seeded in a 24-well culture plate at a density of 10^4^ cells/well. In this experiment, 7F2 osteoblasts were cultivated with mineralization medium (DMEM medium containing 10% FBS, 1% penicillin–streptomycin, 1% L-glutamine, 5 mM β-glycerophosphate and 50 μg/mL ascorbic acid) containing various concentrations of asta-loaded liposomes. The cells were washed twice with PBS and fixed with 75% ethanol for 20 min at 37 °C. Then, the cells were stained with 200 μL of 1% alizarin red S solution for an hour and rinsed with PBS until the supernatants became colorless. The calcified depositions, which appeared in maroon red, were imaged using a microscope (Nicon TI-E) and CCD camera system (SPOT RT3). To quantify the calcium production, 400 μL of 10% *w*/*v* cetylpyridinium chloride (CPC) was added to each well and gently shaken for 10 min to dissolve the calcified depositions. Eventually, the absorbance was measured by an ELISA reader (Tecan, Infinite M200) at a wavelength of 560 nm. The experiments were performed in triplicate.

### 4.15. Quantitative Real-Time PCR

Cells were seeded at a density of 2 × 10^5^ cells in 6 cm dishes and incubated for 24 h. Inflammatory responses in RAW264.7 cells were stimulated with LPS (0.5 μg/mL), and the cells were treated with 0.05 μg/mL asta-loaded liposomes for 24 h.

After treatment, total RNA was extracted using Trizol reagent (RiboZol, AMRESCO, Solon, OH, USA) following the protocol of the manufacturer’s instructions. For reverse transcription, 1 μg of the total RNA was converted to first-strand cDNA using a reverse transcription kit (Promega, Madison, WI, USA). The resulting cDNA (equivalent to 20 ng) was used in a StepOnePlus Real-Time PCR System using FastStart DNA Master-PLUS SYBR Green I (Applied Biosystems, Foster City, CA, USA). The designed primers are shown in Table 1, and all primers used nucleotide sequences present in the PrimerBank database. Each sample was corrected using the mean cycle threshold (CT) value for GAPDH. Relative gene expression was analyzed using the ΔC_T_ method and expressed as fold change (2^−ΔCC^_T_) T relative to the expression values in nonstimulated cells.

### 4.16. Statistical Analysis

Each experiment was performed in triplicate and repeated at least three times with similar results. The values are shown as means ± standard deviations. Data from all experiments were analyzed by Dunn’s post-test using SPSS Version 12 (IBN, New York, NY, USA) and Sigma Plot (San Jose, CA, USA). A p value less than 0.05 was considered statistically significant.

## Figures and Tables

**Table 1:** Physical parameters of liposomal formulations.

Drug Formulation	Particle Size (nm)	PDI Value	Entrapment Efficiency (%)
Empty liposomes	134.35 ± 3.48	0.1502 ± 0.014	--
Asta-loaded liposomes 0.05 μg/mL	123.40 ± 1.83	0.1409 ± 0.027	89.001 ± 0.41
Asta-loaded liposomes 0.25 μg/mL	123.40 ± 3.11	0.1474 ± 0.040	53.905 ± 1.76
Asta-loaded liposomes 0.51 μg/mL	109.68 ± 2.81	0.1661 ± 0.013	43.857 ± 0.81
Asta-loaded liposomes 1.02 μg/mL	127.78 ± 3.44	0.1701 ± 0.032	29.399 ± 0.91

## Data Availability

The data is contained within the article.
